# Disseminated Skeletal Coccidioidomycosis: A Case Report and Comprehensive Review

**DOI:** 10.7759/cureus.91501

**Published:** 2025-09-02

**Authors:** Peter N Rodenko, Colton Herrell, Emily L Rodenko, Josh Elefteratos, Timothy Townsend

**Affiliations:** 1 Medicine, St. George's University School of Medicine, St. George's, GRD; 2 Biology, Trinity University, San Antonio, USA; 3 Internal Medicine, Montefiore Medical Center, Wakefield Campus, Bronx, USA; 4 Radiology, Medical Center Health System, Odessa, USA

**Keywords:** coccidioides, coccidioidomycosis, coccidioidomycosis abscesses, coccidioidomycosis osteomyelitis, coccidioidomycosis young adult male, disseminated coccidioidomycosis, skeletal coccidioidomycosis, soft tissue infections coccidioidomycosis

## Abstract

Coccidioidomycosis, caused by the dimorphic fungi *Coccidioides immitis* and *Coccidioides posadasii*, is endemic to arid regions such as the southwestern United States. Infection typically occurs via inhalation of aerosolized arthroconidia from disrupted soil. In the majority of cases, infection is asymptomatic or results in self-limiting respiratory symptoms; however, dissemination beyond the lungs may occur rarely. Disseminated disease is a rare occurrence, most commonly affecting the skeletal and central nervous systems, with skeletal involvement often manifesting as osteomyelitis in axial locations. This case presents a previously healthy 27-year-old male from an endemic region who developed severe disseminated skeletal coccidioidomycosis without preceding respiratory symptoms. Despite multiple antibiotic treatments prior to admission, his condition progressed to widespread lytic bone lesions and soft tissue abscesses. Neoplastic, rheumatologic, and immunodeficiency-related etiologies were systematically ruled out, and a bone biopsy in conjunction with positive serological findings confirmed *Coccidioides *fungemia. The patient’s risk factors included male sex, Pacific Islander ethnicity, and geographic exposure, all of which are associated with increased susceptibility.

Imaging modalities such as CT and MRI play a critical role in identifying characteristic lytic bone lesions, soft tissue abscesses, and joint space involvement. This case highlights the severity of disseminated coccidioidomycosis presentations and barriers to timely diagnosis in such cases, particularly in the absence of preceding respiratory symptoms. The patient’s favorable response to initial amphotericin B followed by oral azole therapy underscores the importance of early, aggressive antifungal treatment. Beyond this clinical presentation, the report provides a comprehensive review of the disease’s risk factors, diagnostic challenges, and current management strategies. Recognition of this infection’s potential severity, along with a nuanced understanding of its clinical and epidemiologic features, is essential for improving outcomes in affected populations.

## Introduction

Coccidioidomycosis, also known as San Joaquin Valley fever, is caused by the asexual, dimorphic fungi *Coccidioides immitis* and *Coccidioides posadasii*. The disease is endemic to arid regions in the southwestern United States, northern Mexico, and parts of Central and South America. The fungi produce arthroconidia, which are aerosolized from disrupted soil and transform into spherules that release endospores in the lungs. Occasionally, the fungus may disseminate from the lung before the development of adaptive immunity in susceptible individuals. Specifically, alveolar macrophages ingest the spherules or endospores but are unable to eliminate them, allowing the infection to spread hematogenously to other parts of the body [1].

Respiratory *Coccidioides* infections are asymptomatic in approximately 60% of cases or cause self-limiting acute lower respiratory infection [2]. The early stages of pulmonary coccidioidomycosis can often be confused with community-acquired pneumonia, which may lead to public health statistics underreporting the true disease burden [3]. Extrapulmonary dissemination occurs in less than 2% of cases and may result in severe infection that is often fatal if left untreated [3]. There are several subtypes of disseminated infection, including cutaneous, osteoarthritic, and meningeal coccidioidomycosis, characterized by skin lesions and ulcers, painful bone lesions and arthritis, and severe central nervous system (CNS) complications, respectively. Skeletal coccidioidomycosis accounts for approximately half of disseminated infections, most commonly affecting the vertebral spine [4].

The onset of symptoms may be delayed for up to 21 days following exposure to spores, which complicates timely diagnosis and early intervention [2]. A definitive diagnosis for disseminated infection can be achieved through biopsy of the affected tissue or bone. Oral azoles are preferred for mild to moderate local infections, while amphotericin B is reserved for severe cases. This case involves a previously healthy young adult male from an endemic region who presented with severe disseminated skeletal coccidioidomycosis.

## Case presentation

A 27-year-old male presented to the emergency department following transfer from a local emergency department due to multiple painful bone lesions and abscesses. He was transferred to this hospital for workup of suspected metastatic disease with concurrent acute abscess management. The patient reported a remote hospitalization in his home country of Micronesia for an illness consistent with dengue fever. Otherwise, he had no significant past medical history. He denied any personal or family history of cancer. The patient denied tobacco, alcohol, or drug use, and he tested negative for sexually transmitted infections, including HIV. Prior to arrival at the local emergency department, he received multiple courses of outpatient antibiotics, including doxycycline and metronidazole. Upon presentation to the local emergency department prior to transfer, he received intravenous vancomycin, piperacillin-tazobactam, ivermectin, fluids, and antipyretics.

On physical examination, he was cachectic but alert and oriented to time, person, and place. The patient was afebrile and hemodynamically stable, though tachycardic with a heart rate of 122 beats per minute. Multiple abscesses were observed on physical exam in areas over the zygomatic arch, right occipital region, and cervical spine. CT imaging reports from the previous local emergency department showed lytic lesions involving the cervical spine, chest, abdomen, and pelvis, which were concerning for metastatic disease. The neurologic examination was unremarkable, with no focal deficits, and there was no organomegaly. There was right supraclavicular lymphadenopathy on palpation. Relevant labs on admission are shown in Table 1.

**Table 1:** Patient’s lab findings on presentation WBC: white blood cell

Lab	Value	Reference range
WBC (10^9/L)	12.0	4-11
Hemoglobin (g/dL)	8.3	13.4-17.4
Platelets (10^9/L)	653	150-440
Hematocrit (%)	29.0	40-54
Sodium (mEq/L)	137	134-146
Potassium (mEq/L)	3.1	3.5-5
Bicarbonate (mEq/L)	24	24-31
Creatine (mEq/L)	0.6	0.5-1.5
Glucose (mg/L)	109	60-100
Anti-nuclear antibodies	Positive	<1:40
Anti-Smith antibodies	Positive	<1:40

**Figure 1:**
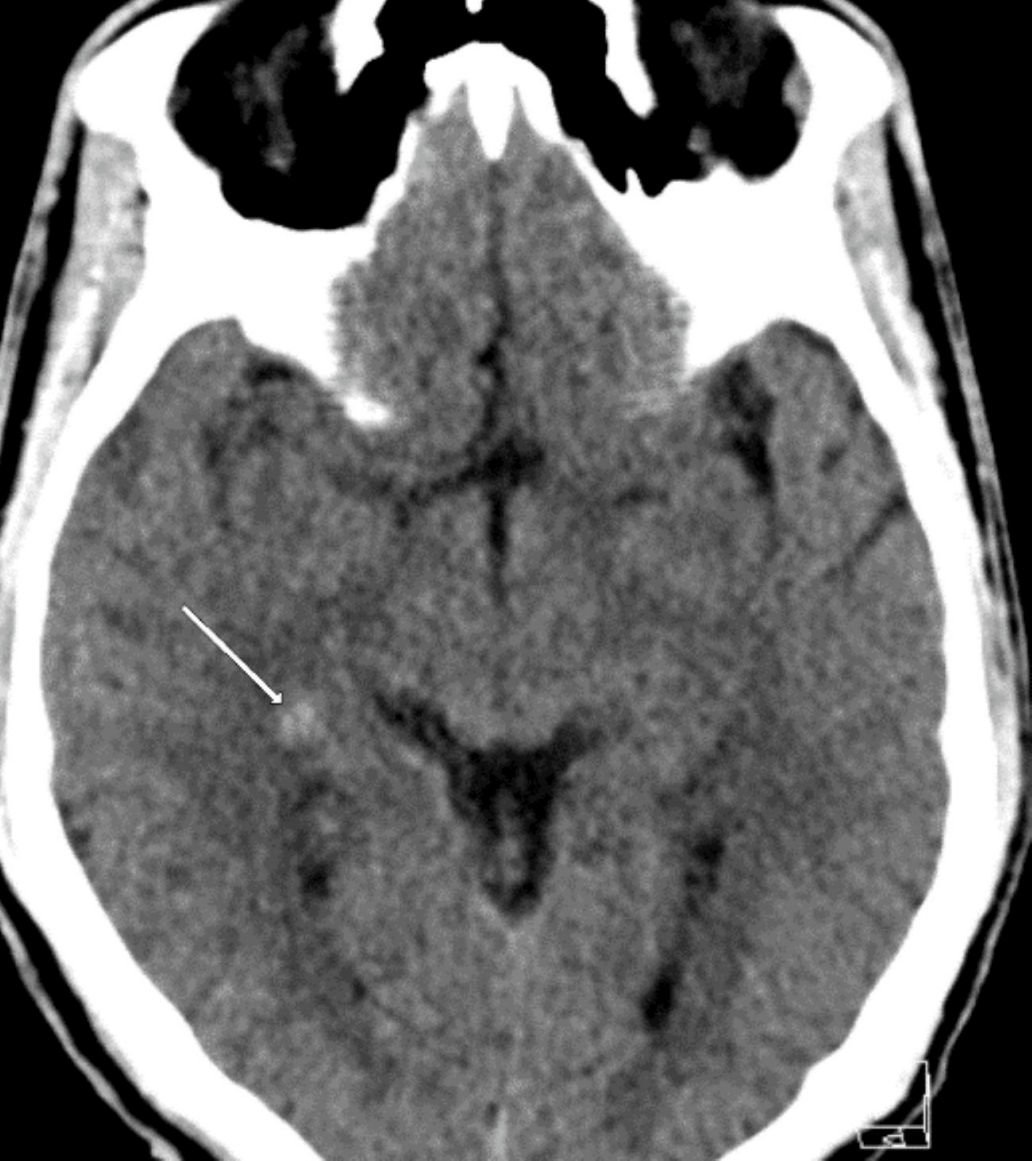
Axial non-contrast enhanced head CT with a small mildly hyperdense lesion adjacent to the right lateral ventricle at the medial right frontoparietal junction (arrow) CT: computed tomography

**Figure 2:**
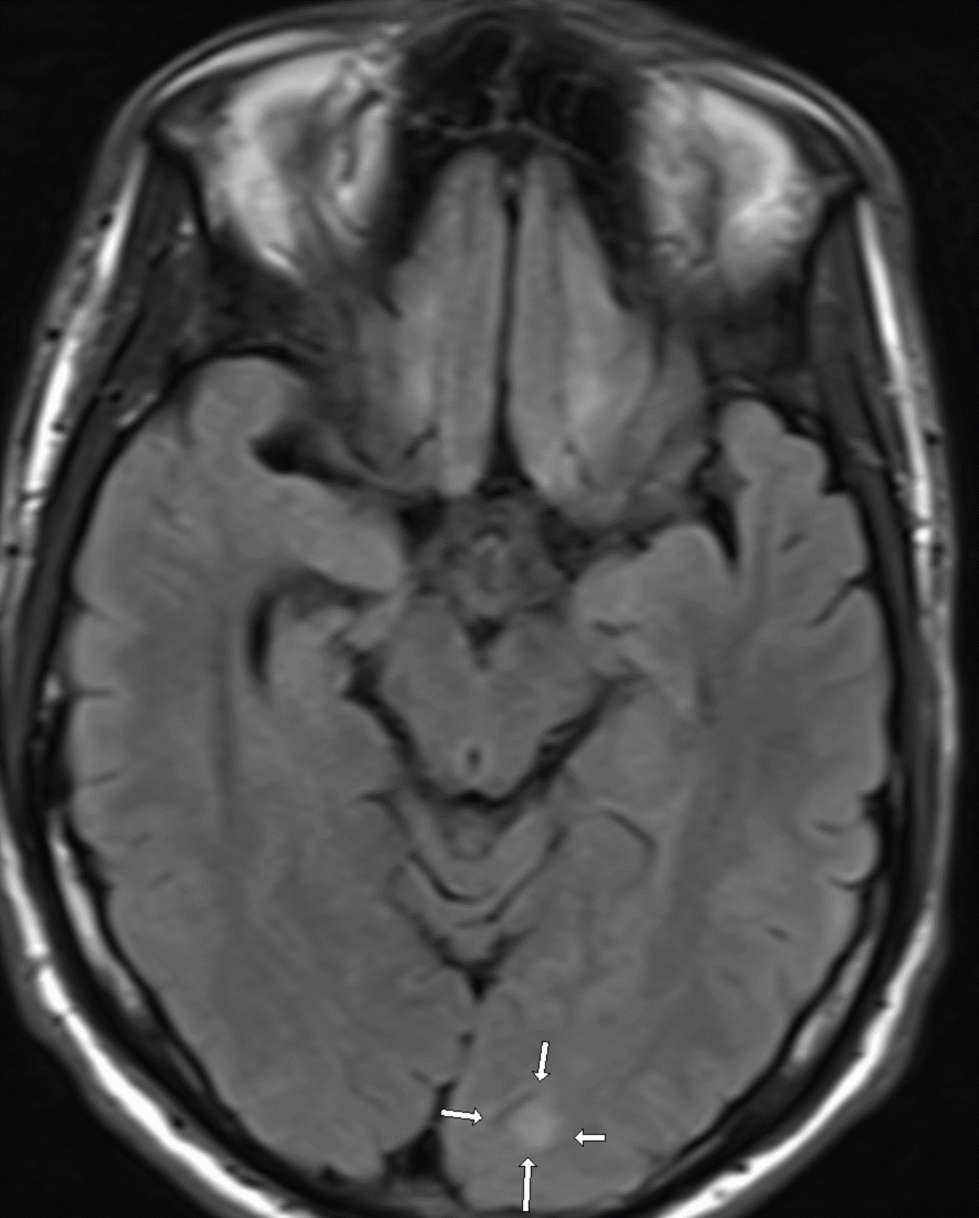
Axial MR FLAIR image of the brain showing a small hyperintense lesion at the gray-white junction in the left occipital lobe (arrows) MR: magnetic resonance, FLAIR: fluid-attenuated inversion recovery

Abnormalities were observed diffusely throughout the body on imaging; as a result, the following figures demonstrate the best representations of notable pathological findings, but they do not encompass all abnormal findings, given the widespread dissemination of the disease. Initial non-contrast CT of the head (Figure 1) revealed a hyperdense lesion adjacent to the right lateral ventricle, raising concern for intraventricular calcification or a mass; MRI was recommended for further evaluation. A subsequent MRI of the brain with and without contrast (Figure 2) showed a small enhancing lesion at the right posterior frontotemporal junction (consistent with Figure 1 findings) and a non-enhancing lesion at the gray-white junction in the left occipital lobe.

**Figure 3:**
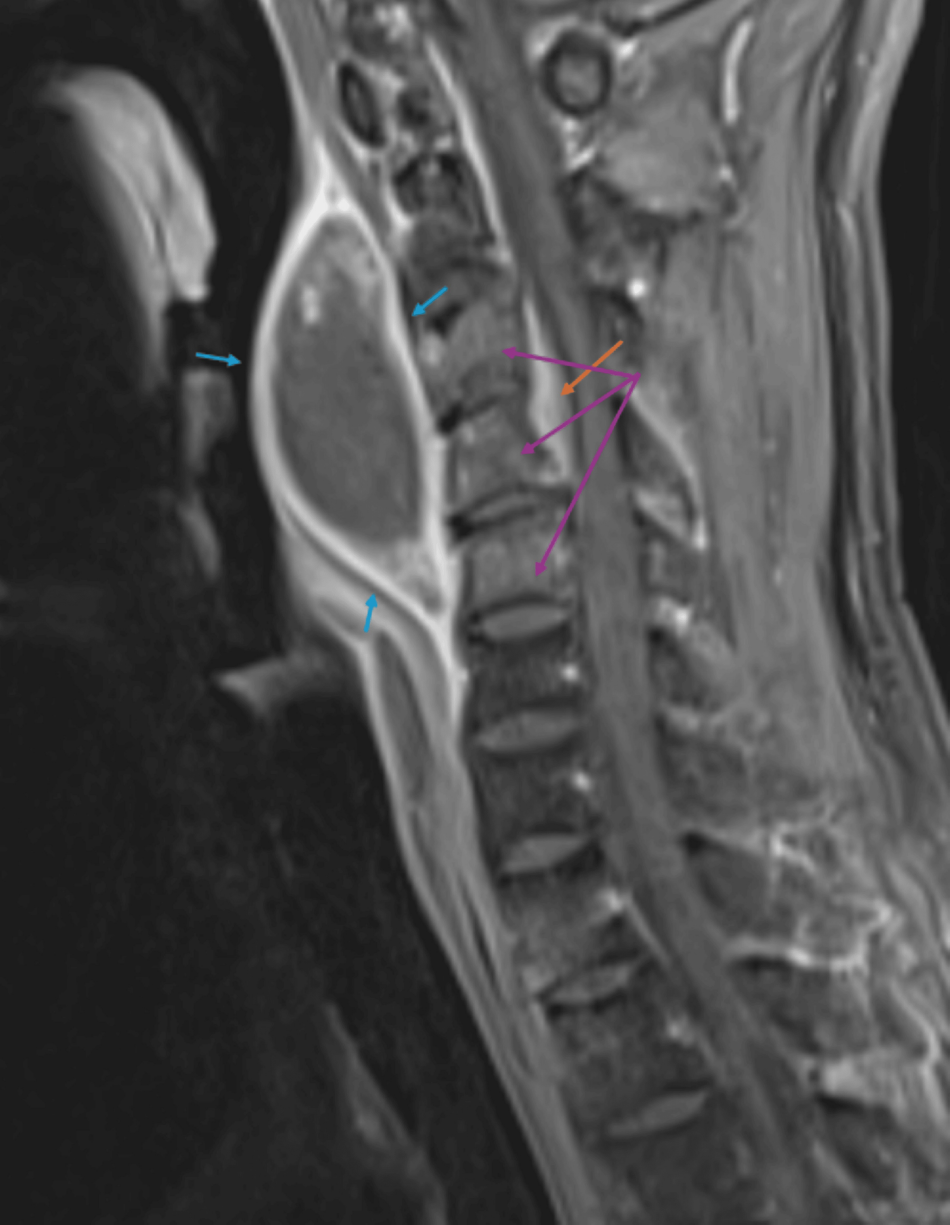
Sagittal contrast-enhanced fat-saturated T1-weighted cervical spine MRI. Findings indicate a homogenous fluid-filled lesion with an enhancing, thick wall, likely representing an abscess between the C1 and C4 vertebrae (blue arrows); enhancing epidural inflammation (orange arrow); and bone marrow enhancement in the C3-C5 vertebral bodies consistent with osteomyelitis (purple arrows) MRI: magentic resonance imaging

**Figure 4:**
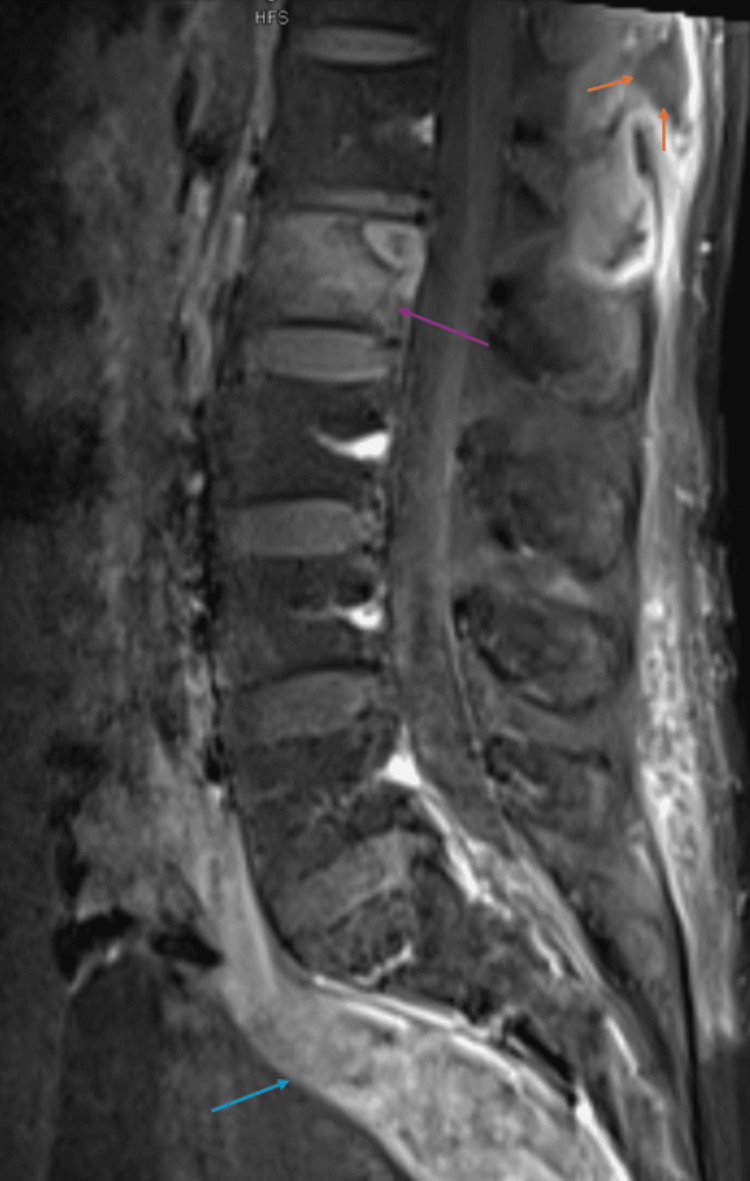
Sagittal contrast-enhanced fat-saturated T1-weighted lumbar spine MRI. Findings demonstrate enhancement of the L2 vertebral body due to osteomyelitis (purple arrow); presacral phlegmonous inflammation (blue arrow); and a small superficial subcutaneous abscess at the level of the thoracolumbar junction (orange arrows) MRI: magnetic resonance imaging

**Figure 5:**
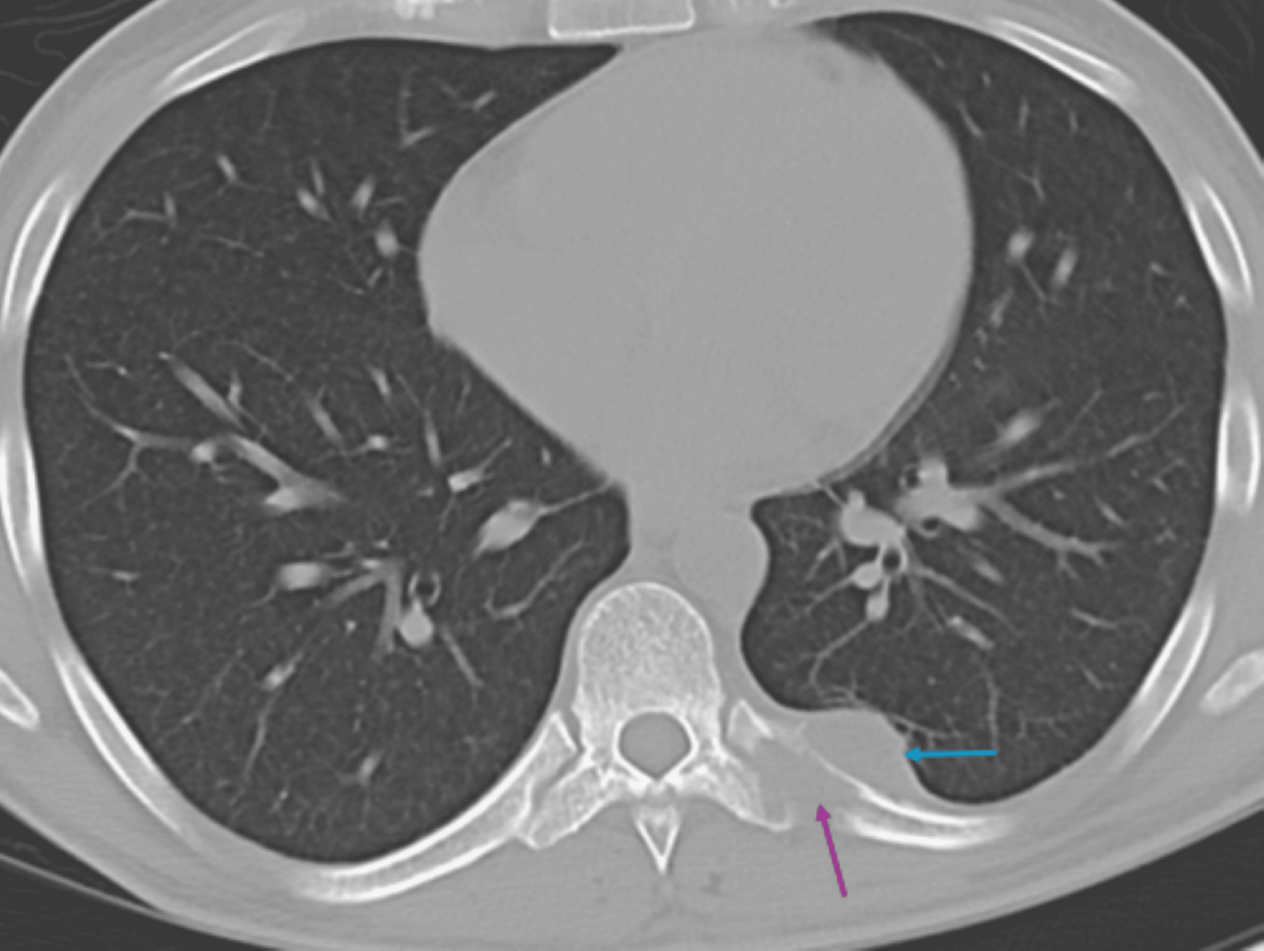
Noncontrast-enhanced axial chest CT demonstrating osteolysis of the posterior left ninth rib and adjacent transverse process consistent with osteomyelitis (purple arrow), with an adjacent pleural-based soft tissue phlegmon (blue arrow) CT: computed tomography

Further MRI of the spine (cervical spine MRI represented by Figure 3; lumbar spine MRI represented by Figure 4) revealed a cervical abscess between the C1 and C4 vertebrae and lumbar phlegmonous changes. There were numerous lytic vertebral bone lesions with associated soft tissue components in the cervical and lumbar spine, consistent with multifocal atypical infection or metastatic disease. There was also an epidural enhancement in the upper cervical region, suggestive of infectious or neoplastic involvement. There was no evidence of spinal cord compression or degenerative changes, and the overall findings favored disseminated infection. CT of the chest without contrast (Figure 5) revealed a pleural-based phlegmon in the left lower lobe with osteomyelitis of the adjacent ninth rib and transverse process of T9.

**Figure 6:**
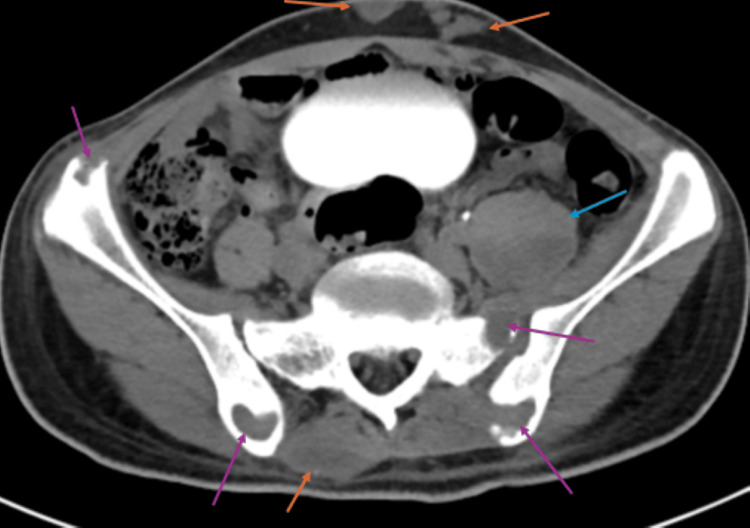
Non-contrast enhanced pelvic CT (with contrast in the urinary tract from a prior exam) demonstrating bilateral iliac destructive bone lucencies due to osteomyelitis, as well as a left sacral destructive lesion also involving the sacroiliac joint (purple arrows); left iliopsoas enlargement due to infection (blue arrow); and subcutaneous infectious lesions (orange arrows) CT: computed tomography

**Figure 7:**
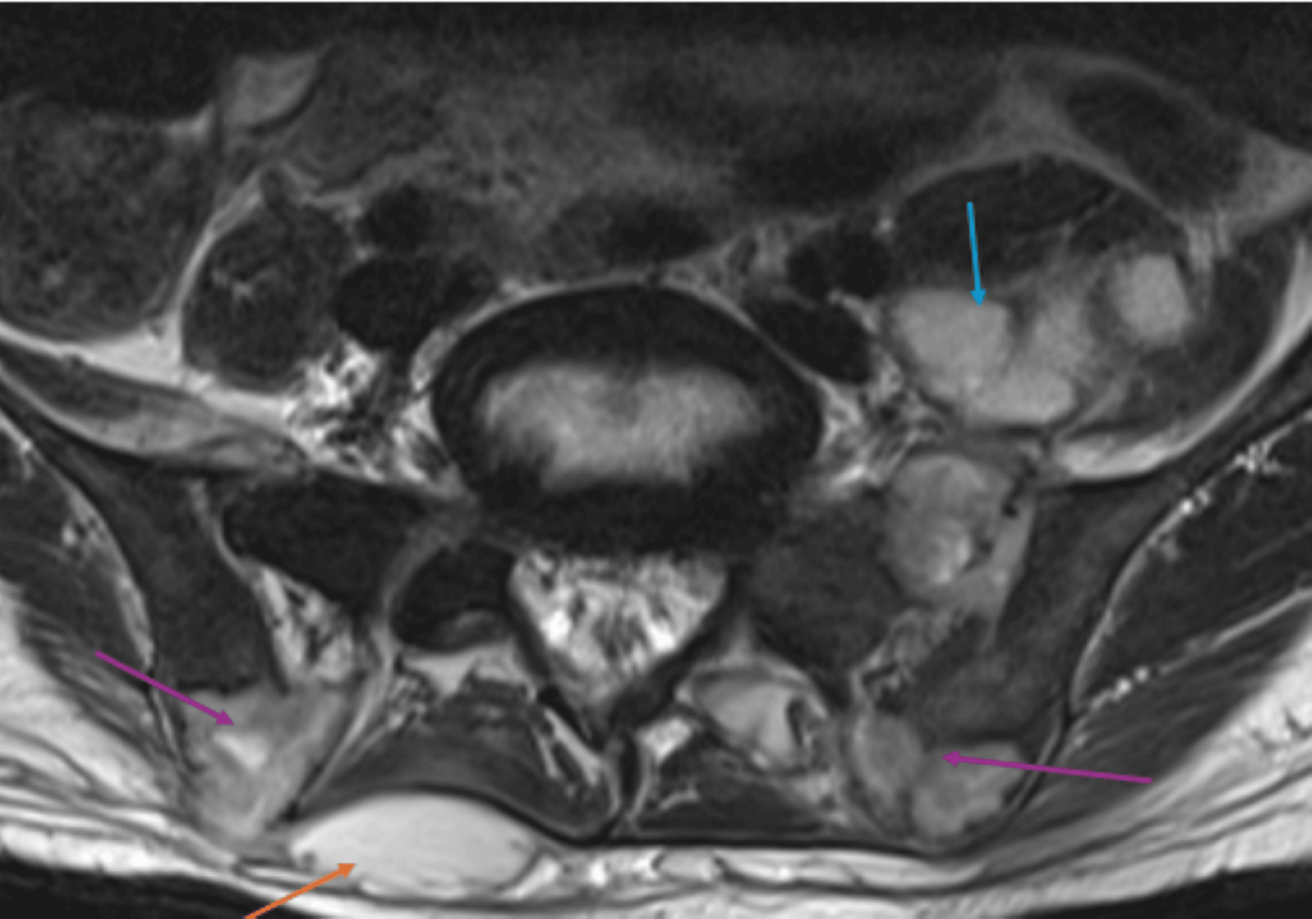
Axial turbo T2-weighted MR image of the upper pelvis demonstrating bilateral iliac bone lesions (purple arrows), left iliopsoas abscess (blue arrow), and posterior subcutaneous abscess (orange arrow) MR: magnetic resonance

A non-contrast CT of the pelvis (Figure 6) showed lytic lesions in the bilateral ilia and left sacrum with involvement of the sacroiliac joint, an enlargement of the left psoas muscle (likely due to infection), and multiple subcutaneous lesions. No masses were seen in the solid abdominal organs. Subsequent MRI (Figure 7) confirmed the CT findings of left psoas abscess and bilateral iliac lesions, as well as another dorsal subcutaneous abscess.

Blood cultures and serologies for viral, bacterial, and common fungal opportunistic infections returned negative. Rheumatology was consulted, and a workup revealed positive antinuclear antibodies and anti-Smith antibodies, raising concern for systemic lupus erythematosus; however, this was ruled out, and these serology findings were attributed to infection-related immune activation. Given the imaging findings, disseminated infection or metastatic malignancy was initially suspected. A biopsy of a right pelvic bone lesion revealed mold-like fungal organisms. Serum testing specific to *Coccidioides* using enzyme-linked immunosorbent assay returned positive for *Coccidioides* antibodies, confirming the diagnosis of disseminated coccidioidomycosis.

The patient was started on intravenous amphotericin B and oral fluconazole, after which they began showing marked clinical improvement. Surgical intervention for abscess drainage in various regions was considered but ultimately deferred, given the patient’s robust response to the antifungal medications and the risk of complications like sinus tracts or fistulas from draining a *Coccidioides* abscess. Following this symptomatic improvement, amphotericin B was discontinued, and the patient was discharged, receiving one year of oral fluconazole. Outpatient follow-up was planned with infectious disease, otolaryngology, and primary care to closely monitor the patient’s status.

## Discussion

This case illustrates a severe case of coccidioidomycosis in a young adult male residing in an endemic region, whose disease course was complicated by widespread fungal dissemination, primarily to the skeletal system. Dissemination is a rare complication following initial respiratory coccidioidomycosis, occurring in approximately 0.5% to 2% of reported cases [3]. This patient likely suffered from an asymptomatic or mild respiratory infection that seeded systemically after an undetermined amount of time, resulting in axial osteomyelitis and abscesses of various musculoskeletal and soft tissue regions. The lack of preceding symptoms or a history of respiratory infection complicates the diagnosis of osteomyelitis in this disease, as it may take months or years for mild or asymptomatic respiratory coccidioidomycosis to reactivate and disseminate [4].

The CNS and skeletal system are among the most common sites of dissemination, with a study of roughly 150 participants reporting CNS involvement in 34% and skeletal involvement in 32% of disseminated cases [5]. Skeletal coccidioidomycosis, especially in axial locations, is often overlooked during clinical investigation in favor of other known causes of skeletal lesions, such as Pott’s disease, neoplasms, or pyogenic spondylitis [4]. Soft tissue spread, as in this case, is a rare manifestation of *Coccidioides* infection and may mimic invasive neoplastic progression on imaging [6]. Given the severity and wide variability of clinical presentations in coccidioidomycosis, accurate diagnosis requires an extensive evaluation of known risk factors, imaging findings, serologic testing, and a systematic exclusion of alternative infectious or neoplastic etiologies.

Although coccidioidomycosis has been well documented since the 1930s [7], its associated risk factors remain variably defined and are still subject to ongoing investigation. Men have been consistently reported to have an increased susceptibility to *Coccidioides*, a finding also supported by animal studies. However, castrated male dogs did not show increased susceptibility to infection, suggesting a hypothesized hormonal influence of testosterone on susceptibility [8]. African American, Filipino, and other Asian/Pacific Islander ethnicities are most commonly cited in literature as having increased risk for *Coccidioides* dissemination and hospitalization. The association between Filipino ethnicity and disease dissemination warrants further investigation, as much of the existing evidence is derived from a limited number of studies, including a 1930s Arizona study likely confounded by a concurrent influx of Filipino migrant farm workers [9]. African and Asian/Pacific Islander ethnicities have been persistently associated with disseminated disease, with studies reporting an 80% chance of disseminated disease in those of African descent versus a 75-80% chance of uncomplicated disease in those with European or admixed American ethnicity [10]. Future studies using genetic subgroups rather than primarily racial/ethnic ones should be pursued to more accurately identify genetic associations since there is marked genetic heterogeneity within races/ethnicities [9].

Extrinsic risk factors for disseminated coccidioidomycosis, as with many fungal infections, are closely linked to impaired immune function. These immune-related risk factors include advanced age (>60 years), tobacco use, and immunocompromised states such as HIV infection, immunosuppressive therapy, diabetes, third-trimester pregnancy, and organ transplantation [5,11]. Occupational exposure represents a standard confounding variable in many studies on coccidiodomycosis risk factors and is arguably the most important. *Coccidioides* mycelia require moisture to grow, but also require long, dry periods to properly desiccate into arthroconidia, making the southwestern United States the ideal climate for growth. Activities that generate dust, such as root farming, construction, or certain hunting activities, as well as natural events like dust storms or earthquakes, are associated with increased rates of infection [12]. The spread of infection through animal reservoirs or vectors has been hypothesized, with kangaroo rats, Arizona pocket mice, bats, armadillos, animal carcasses, or rodent excretions proposed as potential fungal reservoirs, encouraging future zoological and ecological studies [12]. Zoonotic transmission to humans has not been reported to date.

The pathophysiological mechanism of disseminated coccidioidomycosis depends on the transport of the fungus within macrophages through the bloodstream. Due to the small size of inhaled arthroconidia, they can reach the terminal bronchioles, where they replicate, swell, and rupture, leading to the formation of spherules that are too large for adequate neutrophil-mediated destruction or macrophage phagocytosis [9]. Coccidioidomycosis spherules also employ molecular mimicry through the secretion of metalloproteinases, although spherule rupture conversely causes neutrophil chemotaxis [13]. This molecular mimicry may enable *Coccidioides* to enter the bloodstream, masked inside macrophages, thereby facilitating dissemination. To date, there has been no evidence of human-to-human transmission, despite the presence of pulmonary involvement. Genetic susceptibility has been extensively studied, primarily involving genes that affect cellular crosstalk within the interface between the innate and adaptive immune systems. The interleukin-12/interferon gamma pathway has been particularly implicated with predisposatory mutations in IFN-γR1, IL12RB1, and STAT genes [10]. There are more nonspecific genetic mutations that have been implicated, including DECTIN-1, DUOX1, and PLCG2 [10]. Blood type B has previously been reported to be associated with increased susceptibility; however, reanalysis revealed a statistically nonsignificant difference between type B and non-type B individuals, noting that the higher prevalence of type B may reflect its greater frequency among African American and Filipino populations [9].

A collaborative approach is necessary to identify skeletally disseminated coccidioidomycosis in a clinical setting, especially considering this disease may present similarly to other infections or metastatic disease. Assessment of skeletal involvement using both CT and MRI most commonly shows the following: punched-out lytic lesions with circumscribed margins in long and flat bones, permeative bone destruction, soft tissue abscesses with associated osteomyelitis, and skull lesions (the latter of which are uncommon compared to other granulomatous diseases) [14]. Soft tissue involvement can be seen, particularly in the paraspinal soft tissue, where sequelae of abscesses and phlegmon may occur [14]. Using MRI to assess disc space involvement is crucial in vertebral osteomyelitis (which presents in about 25% of disseminated cases), especially since vertebral coccidioidomycosis is more likely to affect the disc space than other granulomatous diseases, making this a relatively specific finding in the appropriate clinical context [14]. The patient in this case exhibited all these imaging findings, which can strongly indicate extrapulmonary coccidioidomycosis in the appropriate clinical setting, even in the absence of confirmatory serology.

The diagnostic workup for disseminated coccidioidomycosis is best performed through serology. Initial serologic testing most frequently involves enzyme immunoassays (EIA), but other techniques, such as immunodiffusion (ID) or complement fixation (CF), can also be used to support the diagnosis [2]. EIA is considered the most sensitive diagnostic testing method. Still, there is evidence to suggest that EIA, ID, and CF testing may be less sensitive than previously thought, particularly in early-stage disease, as described in a retrospective study [15]. This may be partly attributed to the more localized pathology and lower fungal burden of coccidioidomycosis relative to other fungal infections that disseminate more extensively, such as histoplasmosis [15]. Biopsy of bone lesions (usually CT-guided) is the best confirmatory method for diagnosing cases of skeletal involvement, with stains such as hematoxylin-eosin or periodic acid-Schiff being adequate for detection [16].

While treatment may not be necessary in mild pulmonary coccidioidomycosis, disseminated disease requires aggressive antifungal management. The patient in this report was managed according to current guidelines, utilizing amphotericin B acutely in combination with long-term oral azoles, as well as considering surgical intervention to alleviate clinical symptoms. Any patient with symptomatic vertebral involvement due to coccidioidomycosis should be placed on an extensive antifungal regimen for 12-18 months, or lifelong therapy in the case of coccidioidomycosis meningitis [16]. Evidence has suggested that oral azole medications are sufficient alone. However, amphotericin B may be added in severe cases, though it should be used cautiously due to its adverse risk profile [16]. Posaconazole, voriconazole, or IFN-γ may be used in cases refractory to first-line azoles such as fluconazole [17]. Surgical involvement, such as debridement, may also be used in conjunction with medical therapy to limit the spread of infection or alleviate symptoms. The prognosis for extrapulmonary infection is poor; however, early intervention can improve outcomes and reduce the significant economic burden associated with disease diagnosis and management [18]. Adherence to lifelong antifungal therapy can be challenging for these patients, and even proper adherence may not guarantee disease resolution. Patients with severe disease may prevent future disease progression with conservative approaches such as correcting underlying immune dysfunction. Additionally, consultation with medical professionals working in endemic regions is recommended to obtain the most current guidance.

## Conclusions

This case presents a severe, widespread coccidioidomycosis infection in a young adult male from an endemic region, characterized by extensive skeletal and soft tissue involvement. The diagnostic process is guided by a high index of suspicion, with intrinsic risk factors such as male sex, specific ethnicities, and genetic predispositions, as well as extrinsic factors like occupational exposure, playing a key role. Imaging findings, such as circumscribed lytic lesions and soft tissue abscesses, can provide important diagnostic clues, with definitive confirmation achieved through serologic testing and tissue biopsy. Effective management requires prolonged antifungal therapy, typically with oral azoles, and early recognition is essential to improving outcomes and mitigating the significant burden of the disease.
